# Associations between type 1 diabetes and educational outcomes: an Aotearoa/New Zealand nationwide birth cohort study using the Integrated Data Infrastructure

**DOI:** 10.1007/s00125-023-06026-y

**Published:** 2023-10-23

**Authors:** Nicholas Bowden, Rachael Dixon, Vivienne Anderson, Martin de Bock, Alisa Boucsein, Maria Kewene-Edwards, Sheree Gibb, Jesse Kokaua, Octavia Palmer, Ryan Paul, Barry Taylor, Hien Vu, Benjamin J. Wheeler

**Affiliations:** 1 Department of Women’s and Children’s Health, Dunedin School of Medicine, University of Otago, Dunedin, New Zealand; 2 A Better Start National Science Challenge, Auckland, New Zealand; 3 Faculty of Health, University of Canterbury, Christchurch, New Zealand; 4 College of Education, University of Otago, Dunedin, New Zealand; 5 Department of Paediatrics, Te Whatu Ora/Health NZ, Christchurch, New Zealand; 6 Department of Paediatrics, University of Otago, Christchurch, New Zealand; 7 Department of Public Health, University of Otago, Wellington, New Zealand; 8 Centre for Pacific Health, Va’a O Tautai, Health Sciences Division, University of Otago, Dunedin, New Zealand; 9 Waikato Regional Diabetes Service, Hamilton, New Zealand; 10 Te Hutaki Waiora School of Health, University of Waikato, Hamilton, New Zealand; 11 Paediatric Endocrinology, Te Whatu Ora/Health NZ – Southern, Dunedin, New Zealand

**Keywords:** Education, Equity, Paediatrics, School performance, Type 1 diabetes

## Abstract

**Aims/hypothesis:**

Type 1 diabetes is one of the most common chronic diseases of childhood. It is hypothesised that the metabolic and psychosocial consequences of type 1 diabetes may affect educational outcomes; however, existing literature presents conflicting results. This study aimed to assess whether educational outcomes differ for young people with and without type 1 diabetes in Aotearoa/New Zealand (NZ).

**Methods:**

This was a nationwide 9 year birth cohort study of all people born in NZ from 1993 to 2001 using linked administrative data held within the Integrated Data Infrastructure, a national research database containing linked health and non-health data. Educational outcomes of high school attainment, high school attendance and university enrolment were measured from age 13 years until 20 years. Generalised linear regression models with log link and Gaussian distributions were used to compare educational outcomes between those with and those without type 1 diabetes, adjusting for sociodemographic and maternal characteristics.

**Results:**

Of the 442,320 children in the birth cohort, type 1 diabetes was identified in 2058 (0.47%) (mean [SD] age of type 1 diabetes diagnosis 7.7 [3.4] years). Educational outcomes were significantly lower for children with type 1 diabetes than for those without type 1 diabetes, including for any high school qualification (RR 0.97 [95% CI 0.95, 0.99]), university entrance-level high school attainment (RR 0.88 [95% CI 0.84, 0.92]), regular high school attendance (RR 0.91 [95% CI 0.85, 0.97]) and university enrolment (RR 0.93 [95% CI 0.88, 0.98]), even after adjusting for sociodemographic and maternal factors. In addition, educational outcomes were substantially lower for those with post type 1 diabetes diagnosis hospitalisations for diabetic ketoacidosis and hypoglycaemia.

**Conclusions/interpretation:**

In this whole NZ birth cohort study, type 1 diabetes was associated with lower educational outcomes spanning secondary school and into university enrolment. Ongoing efforts to support students with type 1 diabetes are needed, particularly for those with a greater risk profile.

**Graphical Abstract:**

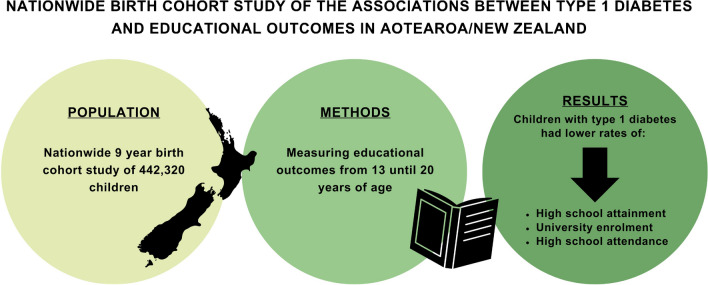

**Supplementary Information:**

The online version contains peer-reviewed but unedited supplementary material available at 10.1007/s00125-023-06026-y.


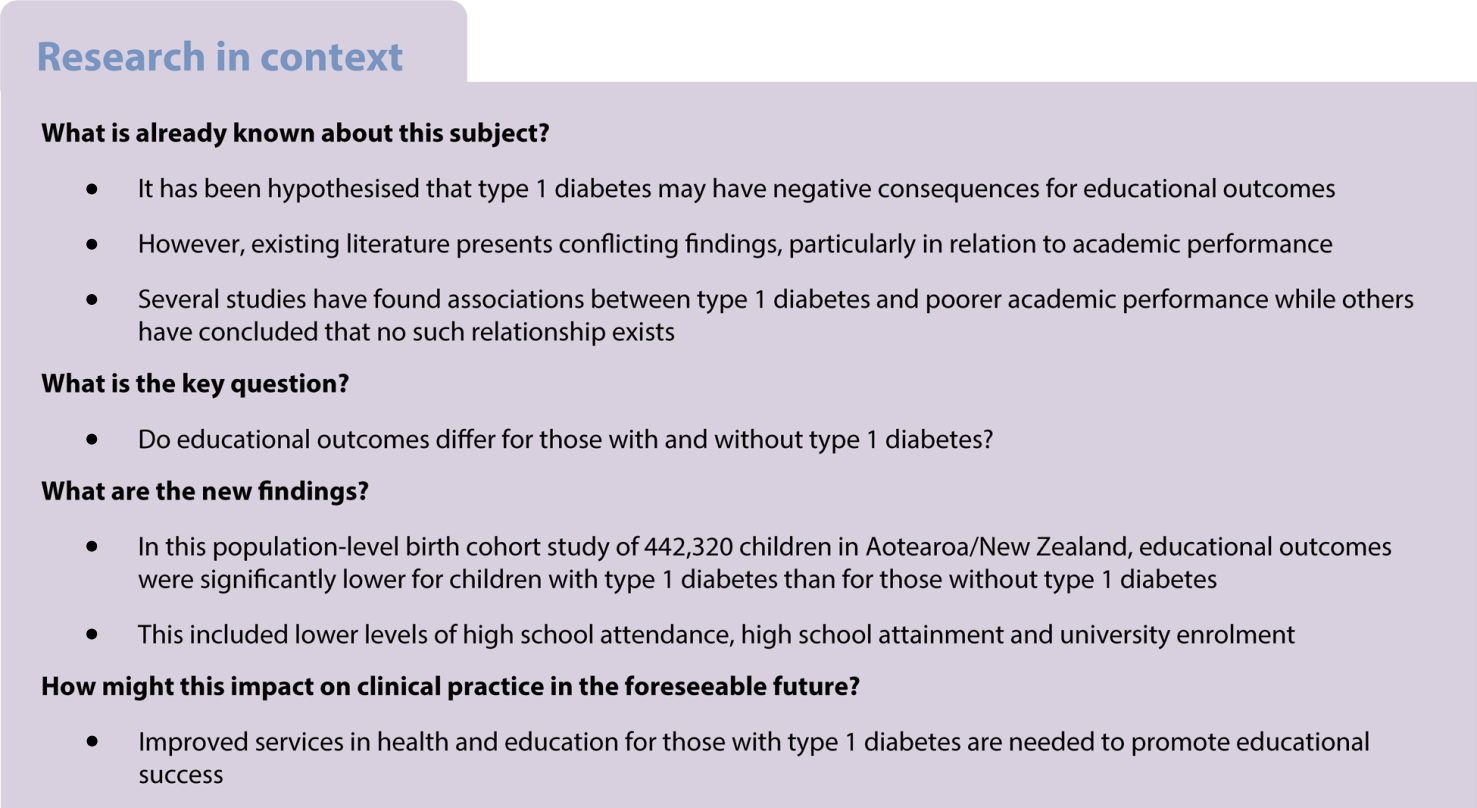


## Introduction

Type 1 diabetes is caused by autoimmune destruction of the insulin-producing cells of the pancreas and is one of the most common chronic diseases of childhood [1]. It is estimated that over 96,000 children (aged <15 years) worldwide develop type 1 diabetes annually [2], with a worldwide prevalence of 9.5 per 10,000 people [3]. For those living with diabetes there are substantial consequences. High glucose levels over a lifetime increase an individual’s risk of diabetes complications [4] and there is evidence that both low and high glucose levels can affect brain function [5–7]. In addition, there are substantial psychological burdens related to type 1 diabetes and its management [8].

Given the numerous and substantial impacts of type 1 diabetes on psychological and cognitive function, it has been hypothesised that type 1 diabetes may have negative consequences for educational outcomes [9, 10]. However, while type 1 diabetes has been associated with school absenteeism [11–13], existing literature pertaining to academic performance presents conflicting findings. Several studies have found associations between type 1 diabetes and poorer academic performance [14–17] while others have found no significant relationship [9, 10, 13, 18–21]. On the other hand, a number of studies have examined student attainment in relation to adverse diabetes outcomes, including diabetic ketoacidosis (DKA) and hypoglycaemia, as well as measures of unhealthy glucose levels, and identified associations with lower academic performance [9, 11, 13, 18]. It has been posited that sample size, selection bias such as excluding unenrolled students from the study population, outcome measurement and availability of modern treatment technologies may be drivers of null results [9, 16, 17].

In Aotearoa/New Zealand (NZ), the Integrated Data Infrastructure (IDI), a national research database containing linked health and non-health data, provides an opportunity to assess educational outcomes for young people with type 1 diabetes using contemporary population-level data [22, 23]. This includes the ability to assess educational outcomes of a multiyear national birth cohort, at several points in their education pathway, including at the points of high-stakes school leaver assessments and university enrolment. This is of particular importance in relation to Māori (the indigenous population of NZ) and Pacific peoples (migrants and their descendants from countries throughout the Pacific region), who are deemed ‘priority learners’ by the NZ Ministry of Education (MoE) [24, 25], but who experience substantial inequities in both education and health [26–28]. IDI data contain large enough samples to enable specific analysis of Māori and Pacific priority populations. Therefore, the objectives of the proposed study were to examine:

- high school attainment, high school attendance and university enrolment (henceforth educational outcomes) among young people with and without type 1 diabetes;
- educational outcomes specifically among Māori and Pacific populations with and without type 1 diabetes; and
- educational outcomes for a subset of children with potentially more disruptive diabetes outcomes (defined as early-onset diabetes prior to 6 years of age, or post type 1 diabetes diagnosis hospitalisation for either DKA or hypoglycaemia).

## Methods

### Study design

This was a national 9 year birth cohort study using data from NZ’s IDI. Databases in the IDI are joined using established record linkage methodologies [29]. Individual data linkage is achieved through a combination of direct unique identifier linking where possible, and probabilistic linking using date of birth, name and sex. The cohort included all those born in NZ between 1993 and 2001 inclusive, identified using Department of Internal Affairs birth registration data. The cohort was restricted to those alive at 20 years of age to permit outcome measurement for the whole population. Those who spent more than 4 years overseas prior to their 13th birthday (typical high school start age and the identification period for type 1 diabetes), or more than 2 years overseas during their high school years, were excluded to improve type 1 diabetes and outcome measurement, as information on diabetes diagnoses or qualifications completed overseas may be unavailable. The study received ethical approval from the University of Otago Human Research Ethics Committee (reference: HD22/046). No further informed consent by participants was required. Reporting of analyses conformed to Reporting of studies Conducted using Observational Routinely-collected health Data (RECORD) guidelines [30].

### Exposure: type 1 diabetes

The type 1 diabetes population was identified using the Te Whatu Ora – Health New Zealand Virtual Diabetes Register (VDR) [31]. The VDR is a list of individuals who are suspected of having diabetes and draws on nationwide data from inpatient events (ICD-10-AM diagnosis codes; https://www.ihacpa.gov.au/health-care/classification/icd-10-amachiacs), outpatient services (e.g. diabetes management services), laboratory tests (e.g. HbA_1c_ tests) and pharmaceutical dispensing (e.g. prescriptions for insulin) [32]. The VDR has a sensitivity of 87%, specificity of 97%, positive predictive value of 82% and negative predictive value of 98% [33]. Individuals were identified with type 1 diabetes if they were first recorded in the VDR before 13 years of age. The VDR does not differentiate type 1 diabetes from type 2 diabetes; however, the vast majority of diabetes cases with onset prior to 13 years of age are type 1 diabetes [34]. To improve the sensitivity of the type 1 diabetes classification, hospitalisation data were used to exclude children with a type 2 diabetes diagnosis code recorded prior to their 13th birthday. A subgroup with early-onset type 1 diabetes (before 6 years of age) was also identified. Lastly, a proxy for more unhealthy glucose levels was constructed among those with type 1 diabetes using hospitalisation data. This indicator was set to 1 if a participant was hospitalised with an ICD-10-AM diagnosis code for type 1 diabetes with ketoacidosis (DKA) (E10.1) or type 1 diabetes with hypoglycaemia (E10.64) prior to the participant’s 13th birthday but at least 30 days after their initial type 1 diabetes diagnosis, and set to 0 otherwise.

### Educational outcomes

#### The New Zealand education system

The NZ education system has three levels: early childhood (birth to school entry), primary and secondary (5–19 years), and further education (higher and vocational). The focus of this study was on primary and secondary education. In NZ, state schools, which are owned and funded by the government, provide free education for children who are NZ citizens or permanent residents between the ages of 5 and 19 years. Schooling is compulsory between the ages of 6 and 16 years. In most schools, children can commence their schooling on the day they turn 5, without having to wait for the start of a new school year. Typically, students remain in school until they reach approximately 18 years. The school education system in NZ comprises 13 year levels. Primary education spans from Years 1–8, and secondary education encompasses Years 9–13.

#### Educational attainment

Educational attainment information was extracted from the MoE student leavers table. The formal measure of a young person’s achievement at high school in NZ is the National Certificate of Educational Achievement (NCEA). Young people usually study for the NCEA in the final 3 years of high school, when they are aged approximately 16–18 years (Levels 1, 2 and 3). The NCEA is a standards-based system consisting of internally (school-based) and externally assessed standards across different school subjects. Students have the flexibility to choose from a wide range of subjects, with English, mathematics and science typically studied, alongside a variety of other options from the eight learning areas of the New Zealand Curriculum. The NCEA is recognised by employers and used by universities and vocational education institutions for selection purposes. In each subject, students’ skills and knowledge are assessed based on specific standards. Students earn credits based on their success meeting the standards; to achieve each NCEA level, 80 credits are required. Specific credits at Level 3 also provide a University Entrance qualification, which enables entry into degree-level tertiary learning. Binary variables reflecting any NCEA attainment (NCEA Level 1, 2 or 3 vs no NCEA attainment) and NCEA Level 3 attainment (vs no NCEA Level 3 attainment) were constructed.

#### Attendance

Data on school attendance were drawn from the MoE attendance table. Attendance data were available from 2015 to 2019 and only for term 2 (the NZ school year is divided into four terms of approximately 10 weeks each). A binary measure of ‘regular attendance’ among enrolled students was created in accordance with MoE definitions [35]. A student was considered to have regular attendance if they attended more than 90% of term 2 half-days. A half-day of attendance is defined as being present for a minimum of 2 h in the morning or the afternoon. Absences were categorised into justified and unjustified in accordance with MoE reporting requirements, and by absence subtype (electronic supplementary material [ESM] Table 1).

#### University enrolment

Data on university enrolment were extracted from the MoE course enrolment table. A binary indicator reflecting any university-level enrolment was constructed and set to 1 if an individual was enrolled in a university-level qualification prior to their 20th birthday and to 0 if no university enrolment was identified. This included enrolment in qualifications at Levels 7–10 from the New Zealand Qualifications and Credentials Framework [36].

### Maternal characteristics

The highest maternal education level was determined using the 2013 and 2018 NZ censuses and MoE data and categorised into four groups: no qualification, secondary school qualification, post-secondary school qualification (not including university-level qualifications, e.g. trade diplomas and certificates) and university-level qualification (e.g. bachelor’s degree, master’s degree and doctor of philosophy degree). The highest level of educational attainment recorded across these datasets was assigned to each individual. A binary variable indicating mother’s birthplace (NZ or elsewhere) and maternal age, measured at the child’s birth and categorised as <20, 20–29, 30–39, 40–49 and ≥50 years, were determined using birth registration data. The number of siblings was determined using birth registration data, counting the number of additional births registered to each mother. Paternal characteristics were not employed as covariates in this analysis because of relatively low linkage rates between children and fathers in the IDI [37].

### Sociodemographic characteristics

Using information captured in the IDI personal details table, sex (male/female), age (in years) and ethnicity were determined. For the current study, we were constrained to utilising data on sex collected using conventional statistical practices for the study period, categorised as male or female. Statistics New Zealand (Stats NZ) introduced an updated statistical standard in 2021, encompassing a more comprehensive understanding of sex and gender identity to tackle concerns such as inclusivity for intersex and transgender communities. Ethnicity was categorised using the NZ Standard Classification as Asian; European and other ethnic groups (see Table 1 for a definition of ‘other ethnic groups’); Māori; Middle Eastern, Latin American or African; and Pacific peoples [38]. Ethnicity was measured using the total concept approach, meaning that individuals could identify with more than one ethnicity. Area level socioeconomic deprivation level was measured using the New Zealand Deprivation Index (NZDep) 2018 [39], linked to residence data from the IDI address notification table. The most recent registered address change prior to an individual’s 13th birthday was used to determine place of residence. If an address change was not registered prior to 13 years of age, the most recent address change within 1 year after their 13th birthday was used. Residence data were also used to determine the urban/rural profile of individuals, a binary indicator using Stats NZ definitions reflecting urban populations of ≥1000 people and rural populations of <1000 people [40].

**Table 1:** Sociodemographic, maternal, and health-related characteristics of the final sample (*N*=442,320) by type 1 diabetes status

Characteristic	With T1D (*n*=2058)	Without T1D (*n*=440,262)
Sex		
Female	996 (48.4)	214,635 (48.8)
Male	1062 (51.6)	225,627 (51.2)
Ethnicity^a^		
Asian	105 (5.1)	25,782 (5.9)
EO^b^	1644 (79.9)	339,279 (77.1)
MELAA	21 (1.0)	4626 (1.1)
Māori	558 (27.1)	135,564 (30.8)
Pacific	270 (13.1)	55,017 (12.5)
Deprivation quintile		
1 (least deprived)	429 (20.8)	83,112 (18.9)
2	393 (19.1)	77,697 (17.6)
3	354 (17.2)	77,631 (17.6)
4	384 (18.7)	81,027 (18.4)
5 (most deprived)	456 (22.2)	104,910 (23.8)
Missing	42 (2.0)	15,885 (3.6)
Residence		
Urban	1755 (85.3)	364,872 (82.9)
Rural	261 (12.7)	59,961 (13.6)
Missing	42 (2.0)	15,429 (3.5)
Mother born in New Zealand		
Yes	1599 (77.7)	341,175 (77.5)
No	459 (22.3)	99,087 (22.5)
Mother's highest qualification		
None	207 (10.1)	44,676 (10.1)
High school	747 (36.3)	151,755 (34.5)
Tertiary certificate or diploma	480 (23.3)	99,477 (22.6)
Bachelor’s degree or higher	405 (19.7)	93,306 (21.2)
Missing	219 (10.6)	51,048 (11.6)
Maternal age (years)		
<20	138 (6.7)	30,819 (7.0)
20–29	975 (47.4)	211,929 (48.1)
30–39	891 (43.3)	186,600 (42.4)
40–49	51 (2.5)	10,002 (2.3)
50+	S	78 (0.0)
Missing	S	834 (0.2)
Number of siblings		
0	243 (11.8)	49,146 (11.2)
1	723 (35.1)	143,838 (32.7)
2	579 (28.1)	118,548 (26.9)
3	297 (14.4)	63,918 (14.5)
4	108 (5.2)	30,633 (7.0)
5+	111 (5.4)	34,176 (7.8)
Clinical		
DKA^c^	276 (13.4)	
Hypoglycaemia^d^	258 (12.5)	
Either DKA or hypoglycaemia	408 (19.8)	
T1D before 6 years of age	705 (34.3)	

Data are presented as *n* (%)

Subtotals may not sum to totals because of rounding as per Stats NZ confidentiality requirements

^a^Percentages do not sum to 100% because individuals could self-identify with multiple ethnic groups

^b^European and other ethnic groups (includes Indigenous American, Mauritian, New Zealander, Seychellois, other South African, and other ethnicity)

^c^Any hospitalisation for post-diagnosis type 1 diabetes with ketoacidosis (≥30 days post type 1 diabetes diagnosis) and prior to 13 years of age

^d^Any hospitalisation for post-diagnosis hypoglycaemia (≥30 days post type 1 diabetes diagnosis) and prior to 13 years of age

MELAA, Middle Eastern, Latin American or African; S, *n*<6; T1d, type 1 diabetes

### Statistical analysis

Observed rates of educational outcomes among the birth cohort were described by sociodemographic subgroup and by type 1 diabetes status overall and for Māori and Pacific peoples. Conventionally employed logistic regression models produce ORs that are biased and inflated estimates of RRs when outcomes of interest are not rare [41]. Therefore, unadjusted and adjusted RRs were generated by type 1 diabetes status for educational outcomes using complete-case generalised linear regression with a log link and Gaussian distribution. A Gaussian distribution was employed to resolve convergence issues, common when using a log link and binomial distribution [42, 43]. Variables in the adjusted models included birth year, sex, ethnicity, deprivation level, urban/rural profile of residence, mother’s birthplace, maternal education level, maternal age and number of siblings in the family. A two-tailed α=0.05 defined significance. This analysis was replicated separately for Māori and for Pacific peoples. In addition, separate analyses were conducted at the population level to examine educational outcomes for those (1) with early-onset type 1 diabetes; (2) hospitalised for DKA or hypoglycaemia; and (3) not hospitalised for DKA or hypoglycaemia or identified with early-onset type 1 diabetes, each compared with those without type 1 diabetes.

A sensitivity analysis was conducted to evaluate the effect of missing educational attainment and attendance data. In accordance with Skipper et al [9], individuals with missing attainment data were assigned the lowest attainment level, no qualification, and enrolled students with missing attendance data were categorised as not having regular attendance. A further sensitivity analysis was conducted excluding students who had ever been enrolled in a specialist school to account for potentially high rates of co-occurring neurodevelopmental conditions (e.g. intellectual disability) among the type 1 diabetes group, which in turn may impact educational outcomes.

## Results

**Fig. 1:**
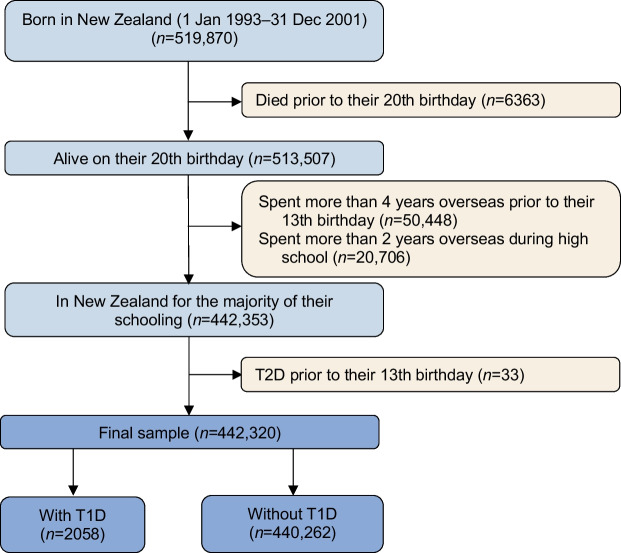
Participant flow chart. T1D, type 1 diabetes; T2D, type 2 diabetes

The 9 year birth cohort contained 519,870 individuals, of whom 77,550 were excluded, yielding a final sample of 442,320 (Fig. 1). Type 1 diabetes was identified in 2058 (0.47%) young people (mean [SD] age of type 1 diabetes diagnosis 7.7 [3.4] years). Among Māori (*N*=136,122), 558 (0.41%) were identified with type 1 diabetes (mean [SD] age of type 1 diabetes diagnosis 9.0 [3.3] years) and among Pacific peoples (*N*=55,287), 270 (0.49%) were identified with type 1 diabetes (mean [SD] age of type 1 diabetes diagnosis 7.4 [3.3] years).

The sociodemographic and maternal characteristics were broadly similar between the group with type 1 diabetes and the group without type 1 diabetes (Table 1). One in five (*n*=408) of the type 1 diabetes group had had a post-diagnosis hospitalisation for either DKA or hypoglycaemia before age 13 years. Approximately one-third (*n*=705) were diagnosed with type 1 diabetes before age 6 years. The characteristics of those with early-onset type 1 diabetes, those hospitalised with DKA/hypoglycaemia and those with neither DKA/hypoglycaemia nor early-onset diabetes, and of Māori and Pacific peoples are presented in ESM Tables 2–4.

Compared with those without type 1 diabetes, children with type 1 diabetes had similar observed rates of any NCEA attainment (82.7% among those with type 1 diabetes vs 82.0% among those without type 1 diabetes) and of university enrolment (39.5% vs 39.9%), but lower observed rates of NCEA Level 3 attainment (43.0% vs 46.3%) and regular attendance (43.1% vs 47.4%) (Table 2). Lower regular attendance among those with type 1 diabetes was mostly driven by higher levels of justified absences, which includes medical absence (ESM Table 5). Among the type 1 diabetes population, those hospitalised with DKA/hypoglycaemia had substantially lower educational outcomes, including lower observed rates of any NCEA attainment (77.2%), NCEA Level 3 attainment (36.0%), regular attendance (33.3%) and university enrolment (32.3%). The educational outcomes for children with early-onset type 1 diabetes and for those without either a hospitalisation for DKA/hypoglycaemia or early-onset type 1 diabetes were generally similar to outcomes in the overall group with type 1 diabetes. Observed educational outcomes for Māori with type 1 diabetes (vs Māori without type 1 diabetes) and for Pacific peoples with type 1 diabetes (vs Pacific peoples without type 1 diabetes) were generally similar.

**Table 2:** Observed educational outcomes among the full sample by type 1 diabetes status, among those with early-onset type 1 diabetes, type 1 diabetes and DKA/hypoglycaemia and type 1 diabetes without DKA/hypoglycaemia, and among Māori and Pacific peoples by type 1 diabetes status

Educational outcome	With T1D (*N*=2058)	Without T1D (*N*=440,262)	With early-onset T1D^a^ (*N*=705)	T1D with DKA/HG^b^ (*N*=408)	T1D without DKA/HG or early onset (*N*=1128)	Māori with T1D (*N*=558)	Māori without T1D (*N*=13,564)	Pacific peoples with T1D (*N*=270)	Pacific peoples without T1D (*N*=55,017)
High school qualification									
Any NCEA	1701 (82.7)	360,840 (82.0)	594 (84.3)	315 (77.2)	933 (82.7)	405 (72.6)	96,264 (71.0)	204 (75.6)	42,108 (76.5)
NCEA Level 3	885 (43.0)	203,637 (46.3)	315 (44.7)	147 (36.0)	489 (43.4)	156 (28.0)	40,917 (30.2)	102 (37.8)	20,223 (36.8)
Missing NCEA data	69 (3.4)	24,909 (5.7)	27 (3.8)	12 (2.9)	39 (3.5)	30 (5.4)	10,692 (7.9)	12 (4.4)	4428 (8.0)
University enrolment^c^									
Yes	570 (39.5)	136,770 (39.9)	144 (38.7)	96 (32.3)	366 (40.8)	96 (24.8)	24,831 (23.6)	45 (27.8)	11,808 (28.2)
No	873 (60.5)	206,379 (60.1)	228 (61.3)	201 (67.7)	531 (59.2)	291 (75.2)	80,556 (76.4)	117 (72.2)	30,027 (71.8)
Attendance (2015–2019)^d^									
Regular attendance^e^	447 (43.1)	83,175 (47.4)	204 (42.8)	66 (33.3)	210 (45.2)	87 (32.6)	15,870 (31.0)	54 (33.3)	7641 (32.7)
Missing attendance data	138 (11.7)	29,238 (14.3)	51 (9.7)	33 (14.3)	69 (12.9)	42 (13.6)	9063 (15.0)	12 (6.9)	2703 (10.4)

Data are presented as *n* (%)

Values may not sum to totals because of rounding as per Stats NZ confidentiality requirements

^a^Type 1 diabetes identified prior to 6 years of age

^b^Any hospitalisation for post-diagnosis type 1 diabetes with ketoacidosis or hypoglycaemia (≥30 days post type 1 diabetes diagnosis) and prior to 13 years of age

^c^The denominators for university enrolment were the number of individuals who turned 20 prior to the end of 2019

^d^The denominators for attendance were the number of individuals enrolled at school any time during the 2015–2019 period

^e^Attendance of 90% or more half-days of term 2 (2015–2019 data)

HG, hypoglycaemia; T1D, type 1 diabetes

**Fig. 2:**
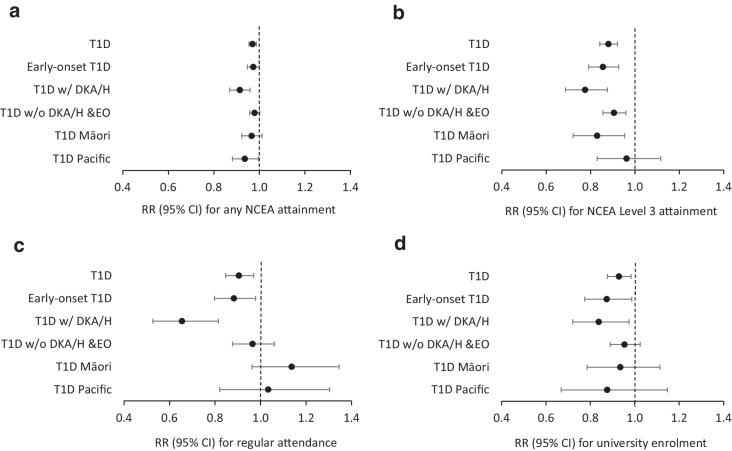
Adjusted RRs (95% CIs) for the association between educational outcomes and T1D status: (**a**) any NCEA attainment; (**b**) NCEA Level 3 attainment; (**c**) regular high school attendance; (**d**) university enrolment. T1D, children with type 1 diabetes vs children without type 1 diabetes; early-onset T1D, children first diagnosed with type 1 diabetes before age 6 years vs children without type 1 diabetes; T1D w/ DKA/H, children with type 1 diabetes and at least one hospitalisation for either DKA or hypoglycaemia before their 13th birthday but at least 30 days after their T1D diagnosis vs children without type 1 diabetes; T1D w/o DKA/H & EO, children with type 1 diabetes and without a hospitalisation for either DKA or hypoglycaemia before their 13th birthday but at least 30 days after their type 1 diabetes diagnosis and children who were not diagnosed with type 1 diabetes before their 6th birthday vs children without type 1 diabetes (EO, European and other ethnic groups [includes Indigenous American, Mauritian, New Zealander, Seychellois, other South African, and other ethnicity]); T1D Māori, Māori children with type 1 diabetes vs Māori children without type 1 diabetes; T1D Pacific, Pacific peoples’ children with type 1 diabetes vs Pacific peoples’ children without type 1 diabetes

Figure 2 displays the adjusted RRs for type 1 diabetes status (and associated 95% CIs) and educational outcomes (see ESM Tables 6–11 for more details). After adjustment for sociodemographic and maternal factors, those with type 1 diabetes had lower educational outcomes across each of the four domains than those without type 1 diabetes (any NCEA attainment: RR 0.97 [95% CI 0.95, 0.99]; NCEA Level 3 attainment: RR 0.88 [95% CI 0.84, 0.92]; regular high school attendance: RR 0.91 [95% CI 0.85, 0.97]; and university enrolment: RR 0.93 [95% CI 0.88, 0.98]) (ESM Table 6). Early-onset type 1 diabetes was associated with even lower NCEA Level 3 attainment (RR 0.86 [95% CI 0.79, 0.93]), regular high school attendance (RR 0.88 [95% CI 0.80, 0.98]) and university enrolment (RR 0.87 [95% CI, 0.78, 0.99]), and similar rates of any NCEA attainment (RR 0.97 [95% CI 0.95, 1.00]) (ESM Table 7). Those with a previous post-diagnosis hospitalisation for DKA/hypoglycaemia had substantively lower educational outcomes across all domains (any NCEA attainment: RR 0.91 [95% CI 0.87, 0.96]; NCEA Level 3 attainment: RR 0.78 [95% CI 0.69, 0.88]; regular high school attendance: RR 0.66 [95% CI 0.53, 0.82]; and university enrolment: RR 0.84 [95% CI, 0.72–0.98]) (ESM Table 8). Young people with type 1 diabetes without either early onset or a hospitalisation for DKA/hypoglycaemia had significantly lower rates of NCEA Level 3 attainment (RR 0.91 [95% CI 0.86, 0.96]), but no other significant associations were found (ESM Table 9). Māori with type 1 diabetes were significantly less likely to achieve NCEA Level 3 than Māori without type 1 diabetes (RR 0.83 [95% CI 0.72, 0.95]), but there were no significant associations with respect to the other outcomes (ESM Table 10). Pacific peoples were significantly less likely to achieve any NCEA qualification than Pacific peoples without type 1 diabetes (RR 0.94 [95% CI 0.88, 0.99]), but no other statistically significant associations were found (ESM Table 11).

Sensitivity analyses that accounted for missing educational outcomes data (ESM Table 12) and that excluded students ever enrolled in a specialist school (ESM Table 13) showed no material difference in findings.

## Discussion

In this nationwide NZ birth cohort study, type 1 diabetes was associated with significantly lower educational outcomes of high school attainment, high school attendance and university enrolment. Educational outcomes were particularly affected among those with type 1 diabetes who, post diagnosis, underwent a hospitalisation for DKA or hypoglycaemia. Among Māori, those with type 1 diabetes experienced significantly lower rates of NCEA Level 3 attainment, whereas, among Pacific peoples, those with type 1 diabetes had significantly lower rates of any NCEA qualification. In the context of conflicting findings in existing literature, the present study further elucidates the relationship between type 1 diabetes and educational outcomes using contemporary data at the population level and exploring outcomes at multiple points in the education system.

The finding that type 1 diabetes is associated with lower educational attainment is consistent with several nationwide studies [14–17] but is in contrast to others [9, 13]. Differences in findings might be due to selection bias. For example, unlike a number of studies with null findings, the present study, and that of Lindkvist et al [17], did not exclude unenrolled students or those in specialist schools [9, 13, 17, 21]. This suggests that such exclusions may mask the impact of type 1 diabetes. Further variability in findings may also be attributable to differences in outcome measurement. In NZ, the NCEA is internally and externally assessed across the year. It is possible that students with type 1 diabetes who experience higher levels of school absence may find achieving internally assessed qualifications (which are often completed over time) particularly difficult. Given that other studies have typically explored outcomes derived from one-off assessments, this may help to explain the negative effect of type 1 diabetes observed in the present study [9, 21, 44]. Cross-country variation in the level and quality of both health support and education support for young people with type 1 diabetes, including variability in the time periods covered and advances in diabetes treatment, may also have influenced the results.

Our results indicating that type 1 diabetes is associated with school absenteeism align with previous research [10–13]. They are also consistent with the lower subsequent educational outcomes observed among the type 1 diabetes group, as school attendance is strongly associated with academic attainment [45]. Similarly, the negative association between type 1 diabetes and university enrolment, as found by Lindkvist et al [17], is also consistent with what one would expect, given lower school attainment levels among the type 1 diabetes group. These findings are concerning given the strong association between university qualifications and later income in NZ [46], and the known link between poor educational outcomes and deleterious consequences in later life such as increased risk of unemployment and crime [47].

The finding that post-diagnosis hospitalisation for DKA or hypoglycaemia was a significant predictor of lower educational outcomes among those with type 1 diabetes is in line with a number of existing studies [9, 11, 17]. Children hospitalised for DKA or hypoglycaemia will generally have less healthy glucose levels, will require more diabetes treatment and will experience a greater psychosocial burden. They may also be from households with fewer resources available, which impacts both health and educational outcomes. Diabetes and glucose variability substantially impact the childhood brain [48–50]. Compounding this effect are the known psychosocial disruptions of childhood diabetes [51–53]. Taken in the context of the now multiple diabetes registry studies highlighting that the majority of children are still not meeting recommended glucose targets [54–56], this study provides a further call to arms for ongoing efforts to continue to improve glucose levels using all proven methods available. Such efforts include improved access and equity to continuous glucose monitoring [57], automated insulin delivery [58] and diabetes psychosocial support, education and nutrition.

As a collective, Māori and Pacific children already experience inequitable educational outcomes compared with non-Māori/non-Pacific children [27, 59]. The findings of this study suggest that, for several educational outcomes, Māori and Pacific peoples with type 1 diabetes are achieving at similar levels to their peers without type 1 diabetes. However, for other outcomes, they indicate that the additional complexity of type 1 diabetes creates multiple disadvantages, widening existing disparities further. This highlights the need for diabetes-related support for students who already face inequitable access to the determinants of health, including the provision of culturally competent and appropriate health and education services [60].

Our findings point to the need for policymakers, school leaders and teachers to be mindful of students’ needs and potential impacts of chronic illness on their access to curriculum content, particularly in relation to high-stakes school leaver assessments. Proactive measures with adequate resourcing to support student achievement and success, including embedding and sustaining a whole-school approach to the promotion of student wellbeing, are required. Initiatives to improve educational outcomes for those with type 1 diabetes are underway in other countries, for example the Diabetes in Schools Program in Australia [61]. However, because they have only recently been implemented, their impact on educational and health outcomes is unknown.

### Strengths and limitations

This study has a number of strengths. It included a large, contemporary, national sample allowing identification of a sizeable group of individuals with type 1 diabetes and enabling analysis of subpopulations including Māori and Pacific peoples, those with early-onset type 1 diabetes and those with a previous hospitalisation for DKA or hypoglycaemia. The data enabled examination of aspects of the pathway through education from high school to university-level study, and adjustment for a range of socioeconomic and maternal measures known to associate with educational outcomes. The study design also accounted for early exit as a result of death or long-term overseas travel.

The study must also be viewed in light of several limitations. The educational attainment outcomes are blunt and do not enable analysis of which areas of learning (e.g. numeracy and literacy) are most affected. Attendance data were available only for term 2 and may not be representative of the full school calendar year. While we included controls for a number of important covariates, other measures associated with educational outcomes such as congenital anomalies, birthweight, gestational age and Apgar score were unable to be accounted for. Moreover, while we excluded, in a sensitivity analysis, students who attended specialist schools, the available data did not allow us to exclude students with special educational needs within the mainstream setting. The impact of omitted variables on study findings is unknown. The VDR is not a formal registry of people with diabetes in NZ; instead, it identifies people who have diabetes by drawing on health service use from multiple datasets. While the VDR is validated and has strong predictive properties (e.g. high sensitivity and specificity), misclassification error may have impacted the study findings [33]. Moreover, the VDR does not readily distinguish between type 1 and type 2 diabetes. However, type 2 diabetes is relatively uncommon in young children (aged <13 years), and those with a type 2 diabetes diagnosis recorded in hospital data were excluded [34]. The data employed in this study do not include measures of HbA_1c,_ which would have been an additional important marker of glucose levels compared with hospitalisations with DKA or hypoglycaemia, which is a less nuanced measure. Lastly, the birth cohort were born mostly in the 1990s and treatment has changed subsequently; however, because of the follow-up time required to observe educational outcomes, the present study was unable to account for newer developments in therapy.

### Implications for future work

Given the inconsistency of the findings in the existing literature, research to better understand the drivers of educational success among those with type 1 diabetes should be prioritised. Among other things, this might include improving the ability of school systems to accommodate chronic illnesses such as type 1 diabetes, and understanding their potential impacts on learning as well as the intersectionality of inequitable access to the social determinants of health and type 1 diabetes. Using temporal data available in the IDI, and more nuanced educational data such as subject-specific attainment information, may help to better elucidate the relationship between type 1 diabetes and educational outcomes. Moreover, further research might also explore other medium- and long-term impacts of type 1 diabetes, such as impacts on employment, income and other forms of continued education. Analysis of the influence of post-pubertal and teenage diagnoses of type 1 diabetes on educational outcomes, because of the substantial psychosocial impacts, also warrants further investigation. Analysis by sex/gender may also provide further insights into the relationship between educational success and type 1 diabetes.

### Conclusion

In this whole NZ cohort study, negative associations between type 1 diabetes and educational outcomes, particularly in those with surrogate markers of glycaemic variability, highlight a further aspect of the profound impact of diabetes on the developing brain and psychosocial development. Ongoing efforts are needed to ensure barrier-free access to education for young people with type 1 diabetes, including school-based supports to enable student achievement and success and equitable outcomes for indigenous populations.

### Supplementary Information

Below is the link to the electronic supplementary material.

ESM Tables (PDF 884 KB)

## Data Availability

The data that support the findings of this study are available from Statistics New Zealand, but restrictions apply to the availability of these data, which were used under licence for the current study and so are not publicly available. The data and code used in this study are, however, available from the authors on reasonable request and with the permission of Statistics New Zealand (see https://www.stats.govt.nz/integrated-data/apply-to-use-microdata-for-research). The data can only be accessed by approved bone fide researchers, for projects that are in the public interest and within a secure accredited data laboratory.

## Abbreviations

DKA: Diabetic ketoacidosis
IDI: Integrated Data Infrastructure
MoE: Ministry of Education
NCEA: National Certificate of Educational Achievement
NZ: Aotearoa/New Zealand
Stats NZ: Statistics New Zealand
VDR: Virtual Diabetes Register
