# Supplementary material for: Associations between type 1 diabetes and educational outcomes: an Aotearoa/New Zealand nationwide birth cohort study using the Integrated Data Infrastructure
Source: Diabetologia. 2023 Oct 23;67(1):62–73. doi: 10.1007/s00125-023-06026-y (PMC10709242; doi:10.1007/s00125-023-06026-y)
Supplement: Supplementary file 1 — ESM Tables (PDF 884 KB) [file 125_2023_6026_MOESM1_ESM.pdf]

Electronic supplementary material

ESM Table 1: School Attendance Codes

Key

P	Present for half day calculations
U	Unjustified absence
J	Justified absence

School Attendance Codes

August 2021

School Code	Classroom	Attendance/absence type	**Explanations** – The following explanations are provided as common reasons why a student may be absent from school. The *Reporting category* column indicates if a School Code is counted as **Present**, **Justified Absence** or **Unjustified Absence** for Ministry reporting purposes. This does not preclude the principal from using discretion over any specific student absence.	Reporting category
P	In class	Present	Student is in their regular class. (This includes supervised study)	P
L	In class	Student is late to class	School policy will determine when this code is used. Eg. School policy may recommend that a student more than 10 minutes late is coded "L". Note this code does **not** contribute to the student's or school's absence or truancy rate.	P
V	In class	Examination or Unsupervised Study – student is on the school-site	Students sitting examinations at school (if the SMS can provide attendance marking during exams). Unsupervised study – school process verifies student is on the school-site. Note that **supervised study** is recorded as a regular timetabled class.	P
I	Not in class	Internal school appointment or activity – Dean, DP, sports administrator, coach, attendance officer etc	This can include students who are out of class for various school appointments including: form teacher, dean, senior management, counsellor, sports administrator, coach, nurse, careers, as well as students on an administration activity such as messenger, collecting attendance etc. It does not include a student who has been removed from his/her regular class and sent to the administration area for disciplinary reasons. This student would be coded P in the class and the code would probably be changed to R by the senior staff member dealing with the student.	P
N	Not in class	On a school-based activity	A school-based (on-site) activity, for example:<br>• cultural/sporting presentation/practice including swimming/athletic sports<br>• one-to-one tuition either as tutor or tutored	P
R	Not in class	Removed (temporarily) from regular class (internal school student isolation)	This code is for students who for a time period had an arrangement for alternative supervision. This may be in the administration corridor or in another teacher's class, instead of the student's regular scheduled class	P
S	Not in class	Sickbay	Student is known to be in the school's sickbay	P
A	Not in class	Attending Alternative Education	The student is not in class, is on the school roll but funded elsewhere	P
H	Not in class	Attending a Health camp/Regional Health School/Residential School	The student is not in class but in an approved environment for which the school is entitled to be funded	P
K	Not in class	Attending a Teen Parent Unit	The student is not in class, is on the school roll but funded elsewhere	P
Y	Not in class	Attending an Activity centre	The student is not in class but in an approved environment for which the school is entitled to be funded	P
Z	Not in class	Secondary Tertiary Programme (including Trades Academies)	The student is participating in a part-time (off-site) approved Secondary Tertiary programme that includes Trades Academies. The school is not entitled to be funded.	P
C	Not in class	Involved in Justice Court proceedings	Under existing legislation this type of absence is deemed to be Present when calculating ½ day summaries	P
D	Not in class	Medical Appointment – doctor or dentist	Current legislation means this type of absence is counted as present for ½ day summaries. There must be documentation verifying the appointment. This code is not to be used for a stay in hospital. Use code "M"	P
F	Not in class	Attending an off-site course/class, or learning from home	The student is not in class but is on a legitimate off-site school-based course. Also used where student is known to be learning from home (for example, during lockdowns).	P
Q	Not in class	Attending an off-site school-organised activity such as trip/camp	A school-organised off-site activity **including overseas:**<br>• school trip (sporting, cultural or academic)<br>• school camp	P
W	Not in class	Work experience	Student is working for a recognised employer as part of their course (Gateway is an example)	P
M	Not in class	Student absent due to short-term illness/medical reasons	Student is at home, or in hospital, because of illness or other medical reason. Depending on school policy a medical certificate may be requested for prolonged illness. eg three days, or as policy requires.	J
J	Not in class	Justified absence – the reason is within the school policy	• Unplanned absences such as a bus breakdown, accident, road closure, extreme weather conditions etc<br>• Planned non-attendance such as national/local representation in a sporting or cultural event in New Zealand or overseas.<br>• Approved absence (also overseas) including bereavement, visiting an ill relative, exceptional family circumstances or a Section 27<br>• A student accompanying, or visiting a family member who is on an overseas posting. (Up to 15 weeks) Eg, military or diplomatic.	J
U	Not in class	Student is Stood down or Suspended	Student is Stood Down or suspended according the conditions of Section 14 of the Education Act 1989. (This code is for the period of the stand down/suspension. It does **not** include the day the stand down was imposed.)	J
T	Not in class	No information provided – truant (or throw-away explanation)	An absence where either no information is provided, or the explanation is trivial (throw-away):<br>• I didn't feel like Maths so I took the period off • I had to finish an important assignment<br>• I went down to the river • I went to the shops • We had a test and I wasn't ready for it	U
E	Not in class	Student is absent. The reason is Explained, but Unjustified	The explanation for the absence is accepted by the school as the reason for the absence. But the reason does not fit within the school's policy as a justifiable reason to take the student off school. (Even though the parents may consider the absence was justified and may have provided a written explanation). Eg, "Molly had to stay home to look after her younger brother". For New Zealand and overseas holidays use code "G".	U
G	Not in class	Holiday during term time	When a student is on a New Zealand or overseas holiday during the school term, the absence is unjustified. A parent's note does not provide justification.	U
O	Not in class	Family reunification	A student is absent due to family reunification (either overseas or within New Zealand) as a result of the opening of the "travel bubble". Counted as Unjustified absence in Ministry reporting. (Still shows as Justified absence in school SMSs.)	U
?	Not in class	Unknown reason (A temporary code)	This is the initial entry for a student not in class and the reason is unknown. It will be edited as relevant information becomes available about the reason for the absence. If required, the SMS can be set by the school to automatically change the '?' code to a T after a configurable number of school days (eg 7)	U
X	Not in class	Exam leave Unsupervised study – student is off-site	Code X is not considered an attendance or an absence and is ignored in the Ministry's attendance calculations. A student coded entirely as code X for a half-day will not be counted at all for that half-day. Note that **supervised study** is recorded as a regular timetabled class.	[N/A]

Source: Ministry of Education (2021)

ESM Table 2: sociodemographic, maternal, and health related characteristics of those with DKA/Hypoglycaemia (N=408), with early onset T1D (N=705), and those with neither DKA/Hypoglycaemia nor early onset T1D (N=1 127)

	Participants, No. (%)		
	**T1D with DKA/Hypoglycaemia T1D**	**Early onset T1D**	**T1D with neither DKA/Hypoglycaemia nor early onset**
Sex			
Female[a]	219 (53.5)	351 (49.8)	522 (46.3)
Male	189 (46.2)	354 (50.2)	606 (53.8)
Ethnicity[b]			
Asian	12 (2.9)	39 (5.5)	57 (5.1)
EO[c]	348 (85.1)	564 (80.0)	894 (79.3)
MELAA[d]	9 (2.2)	9 (1.3)	12 (1.1)
Māori	108 (26.4)	165 (23.4)	330 (29.3)
Pacific	45 (11.0)	102 (14.5)	141 (12.5)
Deprivation quintile			
1 (least deprived)	72 (17.6)	147 (20.9)	255 (22.6)
2	75 (18.3)	153 (21.7)	201 (17.8)
3	75 (18.3)	126 (17.9)	180 (16.0)
4	90 (22.0)	120 (17.0)	216 (19.2)
5 (most deprived)	96 (23.5)	147 (20.9)	246 (21.8)
Missing	..S	12 (1.7)	29 (2.6)
Residence			
Urban	366 (89.5)	615 (87.2)	879 (83.3)
Rural	42 (1033)	78 (11.1)	246 (14.1)
Missing	..S	12 (1.7)	29 (2.6)
Mother born in New Zealand			
Yes	333 (81.4)	540 (76.6)	879 (78.0)
No	78 (19.1)	165 (23.4)	246 (21.8)
Mother's highest qualification			
None	57 (13.9)	69 (9.8)	105 (9.3)
High school	135 (33.0)	267 (37.9)	414 (36.7)
Tertiary certificate or diploma	93 (22.7)	147 (20.9)	276 (24.5)
Bachelor's degree or higher	75 (18.3)	144 (20.4)	219 (19.4)
Missing	49 (12.0)	78 (11.1)	113 (10.0)
Mother's maternal age (years)			
<20	33 (8.1)	51 (7.2)	66 (5.9)
20-29	198 (48.4)	348 (49.4)	522 (46.3)
30-39	162 (39.6)	291 (41.3)	510 (45.3)
40-49	18 (4.4)	15 (2.1)	27 (2.4)
50+	..S	..S	..S
Missing	..S	..S	..S
Number of siblings			
0	51 (12.5)	99 (14.0)	123 (10.9)
1	138 (33.7)	255 (36.2)	396 (35.1)
2	123 (30.1)	186 (26.4)	318 (28.2)
3	54 (13.2)	93 (13.2)	168 (14.9)
4	27 (6.6)	36 (5.1)	57 (5.1)
5+	18 (4.4)	33 (4.7)	66 (5.9)
Clinical			
DKA[e]	276 (67.5)	114 (16.2)	0 (0.0)
Hypoglycaemia[f]	258 (63.1)	132 (18.7)	0 (0.0)
Either DKA or Hypoglycaemia	408 (100.0)	183 (26.0)	0 (0.0)

T1D before 6 years of age	183 (44.7)	705 (100.0)	0 (0.0)

a Values may not sum to totals because of rounding per Statistics New Zealand confidentiality requirements.
b Percentages do not sum to 100% because individuals could self-identify with multiple ethnic groups.
c EO = European and other ethnic groups (includes Indigenous American, Mauritian, New Zealander, Seychellois, other South African, and other ethnicity.
d MELAA = Middle Eastern, Latin American, or African.
e Any hospitalization for post-diagnosis T1D with ketoacidosis (≥30 days post T1D diagnosis) and prior to 13 years of age.
f Any hospitalization for post-diagnosis hypoglycaemia (≥30 days post T1D diagnosis) and prior to 13 years of age.
..S = suppressed due to counts less than 6

ESM Table 3: sociodemographic, family, and health related characteristics among Māori in the final sample (N=136,122) by T1D status

	Participants, No. (%)	
	**T1D (n=558)**	**Without T1D (n=135,564)**
Sex		
Female[a]	279 (50.0)	66,126 (48.8)
Male	279 (50.0)	69,441 (51.2)
Deprivation quintile		
1 (least deprived)	51 (9.1)	10,920 (8.1)
2	72 (12.9)	14,298 (10.5)
3	72 (12.9)	20,136 (14.9)
4	126 (22.6)	28,704 (21.2)
5 (most deprived)	222 (39.8)	54,099 (39.9)
missing	15 (2.7)	4113 (3.0)
Residence		
Urban	468 (86.0)	110,541 (83.5)
Rural	66 (11.8)	18,342 (13.5)
Missing	12 (2.2)	3,984 (2.9)
Mother born in New Zealand		
Yes	510 (91.4)	122,157 (90.9)
No	48 (8.6)	13,407 (9.1)
Mother's highest qualification		
None	75 (13.4)	17,148 (12.6)
High school	189 (33.9)	44,880 (33.1)
Tertiary certificate or diploma	138 (24.7)	34,818 (25.7)
Bachelor’s degree or higher	90 (16.1)	21,750 (16.0)
Missing	66 (11.8)	16,968 (12.5)
Mother’s maternal age (years)		
<20	75 (13.4)	18,936 (14)
20-29	315 (56.5)	75,666 (55.8)
30-39	159 (28.5)	38,376 (28.3)
40-49	12 (2.2)	2307 (1.7)
50	..S	24 (0.0)
Missing	..S	255 (0.2)
Number of siblings		
0	69 (12.4)	12,006 (8.9)
1	114 (20.4)	29,016 (21.4)
2	147 (26.3)	32,574 (24.0)
3	111 (19.9)	25,146 (18.5)
4	54 (9.7)	15,825 (11.7)
5+	63 (11.3)	21,000 (15.5)
Clinical		
DKA[b]	87 (15.6)	
Hypoglycaemia[c]	60 (10.8)	
Either DKA or Hypoglycaemia	108 (19.4)	
T1D before 6 years of age	165 (29.6)	

a Values may not sum to totals because of rounding per Statistics New Zealand confidentiality requirements.
b Any hospitalization for post-diagnosis T1D with ketoacidosis (≥30 days post T1D diagnosis) and prior to 13 years of age.
c Any hospitalization for post-diagnosis hypoglycaemia (≥30 days post T1D diagnosis) and prior to 13 years of age.
..S = suppressed due to counts less than 6

ESM Table 4: sociodemographic, family, and health related characteristics among Pacific peoples in the final sample (N=55,287) by T1D status

	Participants, No. (%)	
	T1D (n=270)	Without T1D (n=55 017)
Sex		
Female[a]	132 (48.9)	27,114 (49.3)
Male	138 (51.1)	27,903 (50.7)
Deprivation quintile		
1 (least deprived)	15 (5.6)	2976 (5.4)
2	18 (6.7)	4284 (7.8)
3	27 (10.0)	6219 (11.3)
4	66 (24.4)	10,971 (19.9)
5 (most deprived)	141 (52.2)	29,508 (53.6)
missing	..S	1059 (1.9)
Residence		
Urban	258 (95.6)	52,122 (94.7)
Rural	9 (3.3)	1875 (3.4)
Missing	..S	1020 (1.9)
Mother born in New Zealand		
Yes	147 (54.4)	27,882 (50.7)
No	123 (45.6)	27,135 (49.3)
Mother's highest qualification		
None	36 (13.3)	7113 (12.9)
High school	93 (34.4)	18,789 (34.2)
Tertiary certificate or diploma	57 (21.1)	11,259 (20.5)
Bachelor’s degree or higher	45 (16.7)	7308 (13.3)
Missing	39 (14.4)	10,548 (19.2)
Mother’s maternal age (years)		
<20	33 (12.2)	6198 (11.3)
20-29	141 (52.2)	29,433 (53.5)
30-39	78 (28.9)	17,799 (32.4)
40-49	18 (6.7)	1410 (2.6)
50	..S	30 (0.1)
Missing	..S	147 (0.3)
Number of siblings		
0	33 (12.2)	6645 (12.1)
1	45 (16.7)	10,227 (18.6)
2	69 (25.6)	11,916 (21.7)
3	60 (22.2)	10,545 (19.2)
4	24 (8.9)	6849 (12.4)
5+	36 (13.3)	8835 (16.1)
Clinical		
DKA[b]	36 (13.3)	
Hypoglycaemia[c]	30 (11.1)	
Either DKA or Hypoglycaemia	42 (15.6)	
T1D before 6 years of age	102 (37.8)	

a Values may not sum to totals because of rounding per Statistics New Zealand confidentiality requirements.
b Any hospitalization for post-diagnosis T1D with ketoacidosis (≥30 days post T1D diagnosis) and prior to 13 years of age.
c Any hospitalization for post-diagnosis hypoglycaemia (≥30 days post T1D diagnosis) and prior to 13 years of age.
..S = suppressed due to counts less than 6

ESM Table 5: Breakdown of attendance and absence information among those with T1D

	With T1D					
	**2015**	**2016**	**2017**	**2018**	**2019**	**Total**
Present %	86.5	85.3	84.2	82.6	79.4	84.9
Unjustified absence %	5.5	6.3	7.3	8.3	11.3	6.7
T (no information provided) %	2.9	3.4	4.1	5.1	7.1	3.7
E (explained but unjustified) %	1.2	1.4	1.6	1.5	2.0	1.4
G (holiday during term) %	0.4	0.4	0.6	0.4	0.5	0.4
? (unknown) %	1.0	1.0	0.9	1.3	1.7	1.1
Justified absence %	8.0	8.5	8.6	9.1	9.3	8.4
M (medical) %	6.4	6.8	7.0	6.7	7.0	6.7
J (justified) %	1.5	1.6	1.5	2.4	2.2	1.7
U (stood down/suspended) %	0.1	0.1	0.0	0.1	0.1	0.1
	**Without T1D**					
Present %	88.0	87.1	85.7	85.0	82.7	86.8
Unjustified absence %	5.7	6.4	7.0	7.6	8.7	6.4
T (no information provided) %	3.1	3.6	4.3	4.6	5.3	3.7
E (explained but unjustified) %	1.3	1.3	1.4	1.5	1.7	1.3
G (holiday during term) %	0.3	0.4	0.4	0.5	0.6	0.4
? (unknown) %	0.9	1.0	0.9	1.0	1.2	1.0
Justified absence %	6.4	6.5	7.2	7.4	8.6	6.8
M (medical) %	4.8	4.9	5.6	5.4	6.4	5.1
J (justified) %	1.5	1.5	1.6	1.9	2.1	1.6
U (stood down/suspended) %	0.1	0.1	0.1	0.0	0.0	0.1

ESM Table 6: Unadjusted and adjusted risk ratios of educational outcomes on T1D status using complete-case generalized linear regression with a log link and gaussian distribution

	Any NCEA Attainment		NCEA 3 Attainment		School Attendance		Tertiary Enrolment	
	RR (95% CI)	ARR (95% CI)	RR (95% CI)	ARR (95% CI)	RR (95% CI)	ARR (95% CI)	RR (95% CI)	ARR (95% CI)
T1D Status								
No	1 (reference)		1 (reference)		1 (reference)		1 (reference)	
Yes	0.979 (0.961, 0.996)	0.970 (0.954, 0.987)	0.891 (0.848, 0.936)	0.881 (0.842, 0.921)	0.907 (0.846, 0.973)	0.905 (0.846, 0.969)	0.986 (0.925, 1.051)	0.929 (0.877, 0.984)
Sex								
Female	1 (reference)		1 (reference)		1 (reference)		1 (reference)	
Male		0.961 (0.958, 0.963)		0.767 (0.763, 0.772)		1.126 (1.115, 1.136)		0.716 (0.711, 0.721)
Ethnicity								
Asian		1.052 (1.047, 1.056)		1.234 (1.222, 1.247)		1.274 (1.251, 1.297)		1.332 (1.314, 1.351)
EO		1.044 (1.040, 1.049)		1.017 (1.007, 1.028)		1.061 (1.043, 1.080)		1.067 (1.053, 1.081)
MELAA		0.969 (0.956, 0.982)		0.924 (0.895, 0.953)		0.995 (0.939, 1.053)		0.906 (0.869, 0.946)
Māori		0.940 (0.937, 0.943)		0.807 (0.800, 0.815)		0.717 (0.705, 0.728)		0.771 (0.761, 0.781)
Pacific		1.046 (1.041, 1.050)		1.008 (0.997, 1.020)		0.819 (0.801, 0.837)		0.961 (0.945, 0.977)
Deprivation								
1 (least deprived)	1 (reference)		1 (reference)		1 (reference)		1 (reference)	
2		0.989 (0.986, 0.991)		0.930 (0.924, 0.937)		0.959 (0.947, 0.971)		0.926 (0.918, 0.935)
3		0.972 (0.969, 0.975)		0.864 (0.857, 0.871)		0.914 (0.901, 0.926)		0.856 (0.847, 0.865)
4		0.952 (0.949, 0.955)		0.791 (0.784, 0.798)		0.861 (0.848, 0.875)		0.779 (0.770, 0.789)
5 (most deprived)		0.903 (0.899, 0.907)		0.696 (0.689, 0.704)		0.772 (0.758, 0.787)		0.648 (0.638, 0.658)
Rural residential location								
No	1 (reference)		1 (reference)		1 (reference)		1 (reference)	
Yes		1.010 (1.007, 1.013)		0.939 (0.931, 0.947)		1.064 (1.05, 1.077)		0.874 (0.864, 0.884)
Mother NZ born								
No	1 (reference)		1 (reference)		1 (reference)		1 (reference)	
Yes		1.022 (1.020, 1.025)		1.053 (1.046, 1.061)		1.043 (1.03, 1.056)		1.065 (1.055, 1.074)
Mother highest qual								

No qualification	1 (reference)	1 (reference)	1 (reference)	1 (reference)
School qualification	1.114 (1.107, 1.120)	1.430 (1.408, 1.452)	1.189 (1.163, 1.215)	1.521 (1.489, 1.554)
Tertiary certificate/diploma	1.132 (1.125, 1.138)	1.528 (1.504, 1.552)	1.200 (1.173, 1.228)	1.725 (1.688, 1.763)
University degree or higher	1.169 (1.163, 1.176)	1.828 (1.800, 1.857)	1.299 (1.271, 1.328)	2.212 (2.165, 2.260)
Mother's maternal age (years)				
<20	0.921 (0.915, 0.928)	0.687 (0.672, 0.702)	0.782 (0.760, 0.805)	0.632 (0.612, 0.653)
20-29	1 (reference)	1 (reference)	1 (reference)	1 (reference)
30-29	1.028 (1.026, 1.031)	1.192 (1.185, 1.199)	1.084 (1.073, 1.095)	1.212 (1.202, 1.221)
40-49	1.020 (1.013, 1.027)	1.193 (1.174, 1.213)	1.033 (1.000, 1.068)	1.249 (1.223, 1.276)
50+	0.904 (0.773, 1.058)	0.752 (0.520, 1.087)	0.986 (0.557, 1.746)	0.864 (0.507, 1.473)
Number of siblings				
0	1 (reference)	1 (reference)	1 (reference)	1 (reference)
1	1.017 (1.014, 1.021)	1.059 (1.050, 1.069)	1.085 (1.067, 1.103)	1.049 (1.036, 1.062)
2	1.007 (1.003, 1.011)	1.033 (1.023, 1.043)	1.069 (1.050, 1.088)	1.020 (1.007, 1.033)
3	0.976 (0.972, 0.981)	0.955 (0.944, 0.967)	0.999 (0.978, 1.021)	0.920 (0.906, 0.935)
4	0.931 (0.924, 0.937)	0.824 (0.809, 0.840)	0.878 (0.851, 0.906)	0.779 (0.760, 0.799)
5+	0.848 (0.841, 0.856)	0.707 (0.692, 0.723)	0.777 (0.750, 0.806)	0.628 (0.609, 0.648)
N	360,351	360,351	158,547	289,731

*Adjusted for birth year, sex, ethnicity, deprivation level, urban/rural profile of residence, maternal education level, maternal born in NZ status, maternal age, and number of siblings.

ESM Table 7: Unadjusted and adjusted risk ratios of educational outcomes on early onset T1D status using complete-case generalized linear regression with a log link and gaussian distribution

	Any NCEA Attainment		NCEA 3 Attainment		School Attendance		Tertiary Enrolment	
	RR (95% CI)	ARR (95% CI)	RR (95% CI)	ARR (95% CI)	RR (95% CI)	ARR (95% CI)	RR (95% CI)	ARR (95% CI)
T1D Status								
No	1 (reference)		1 (reference)		1 (reference)		1 (reference)	
Yes	0.988 (0.961, 1.016)	0.973 (0.947, 1.000)	0.893 (0.823, 0.968)	0.856 (0.791, 0.927)	0.907 (0.818, 1.006)	0.883 (0.798, 0.978)	0.975 (0.858, 1.108)	0.874 (0.775, 0.986)
Sex								
Female	1 (reference)		1 (reference)		1 (reference)		1 (reference)	
Male		0.960 (0.958, 0.963)		0.767 (0.763, 0.772)		1.125 (1.115, 1.136)		0.716 (0.711, 0.722)
Ethnicity								
Asian		1.052 (1.047, 1.056)		1.234 (1.222, 1.247)		1.273 (1.250, 1.296)		1.333 (1.315, 1.351)
EO		1.044 (1.040, 1.049)		1.018 (1.008, 1.028)		1.061 (1.043, 1.079)		1.068 (1.053, 1.082)
MELAA		0.968 (0.955, 0.982)		0.924 (0.896, 0.953)		0.994 (0.939, 1.053)		0.906 (0.868, 0.945)
Māori		0.940 (0.937, 0.943)		0.807 (0.800, 0.815)		0.716 (0.705, 0.728)		0.771 (0.761, 0.781)
Pacific		1.046 (1.041, 1.051)		1.009 (0.997, 1.021)		0.819 (0.801, 0.837)		0.961 (0.945, 0.977)
Deprivation								
1 (least deprived)	1 (reference)		1 (reference)		1 (reference)		1 (reference)	
2		0.989 (0.987, 0.991)		0.930 (0.924, 0.937)		0.959 (0.947, 0.971)		0.926 (0.918, 0.935)
3		0.972 (0.969, 0.975)		0.864 (0.857, 0.871)		0.914 (0.901, 0.926)		0.855 (0.846, 0.864)
4		0.952 (0.949, 0.955)		0.791 (0.784, 0.798)		0.861 (0.848, 0.875)		0.779 (0.770, 0.789)
5 (most deprived)		0.903 (0.899, 0.907)		0.696 (0.688, 0.704)		0.772 (0.758, 0.787)		0.648 (0.638, 0.658)
Rural residential location								
No	1 (reference)		1 (reference)		1 (reference)		1 (reference)	
Yes		1.010 (1.007, 1.013)		0.939 (0.931, 0.947)		1.064 (1.050, 1.077)		0.874 (0.864, 0.885)
Mother NZ born								
No	1 (reference)		1 (reference)		1 (reference)		1 (reference)	
Yes		1.022 (1.020, 1.025)		1.053 (1.046, 1.061)		1.043 (1.030, 1.056)		1.065 (1.055, 1.074)
Mother highest qual								

No qualification	1 (reference)	1 (reference)	1 (reference)	1 (reference)
School qualification	1.114 (1.107, 1.120)	1.429 (1.407, 1.452)	1.189 (1.163, 1.215)	1.521 (1.489, 1.554)
Tertiary certificate/diploma	1.132 (1.125, 1.138)	1.527 (1.503, 1.551)	1.200 (1.173, 1.227)	1.725 (1.689, 1.763)
University degree or higher	1.169 (1.163, 1.176)	1.827 (1.799, 1.856)	1.299 (1.271, 1.329)	2.212 (2.165, 2.260)
Mother's maternal age (years)				
<20	0.921 (0.914, 0.928)	0.688 (0.673, 0.703)	0.783 (0.761, 0.806)	0.633 (0.613, 0.654)
20-29	1 (reference)	1 (reference)	1 (reference)	1 (reference)
30-29	1.028 (1.026, 1.030)	1.192 (1.185, 1.199)	1.084 (1.073, 1.095)	1.212 (1.202, 1.221)
40-49	1.020 (1.013, 1.027)	1.193 (1.174, 1.213)	1.033 (1.000, 1.068)	1.249 (1.223, 1.275)
50+	0.904 (0.773, 1.058)	0.751 (0.520, 1.087)	0.986 (0.557, 1.746)	0.864 (0.507, 1.473)
Number of siblings				
0	1 (reference)	1 (reference)	1 (reference)	1 (reference)
1	1.017 (1.014, 1.021)	1.060 (1.050, 1.070)	1.085 (1.067, 1.104)	1.049 (1.036, 1.062)
2	1.007 (1.003, 1.011)	1.034 (1.024, 1.044)	1.070 (1.051, 1.089)	1.020 (1.007, 1.033)
3	0.976 (0.972, 0.981)	0.956 (0.944, 0.967)	1.000 (0.978, 1.021)	0.921 (0.906, 0.935)
4	0.931 (0.924, 0.938)	0.825 (0.810, 0.840)	0.878 (0.851, 0.906)	0.780 (0.761, 0.800)
5+	0.848 (0.841, 0.856)	0.707 (0.692, 0.723)	0.777 (0.749, 0.806)	0.628 (0.609, 0.648)
N	359,196	359,196	158,043	288,792

*Adjusted for birth year, sex, ethnicity, deprivation level, urban/rural profile of residence, maternal education level, maternal born in NZ status, maternal age, and number of siblings.

ESM Table 8: Unadjusted and adjusted risk ratios of educational outcomes on T1D with hospitalization for DKA/hypoglycaemia status using complete-case generalized linear regression with a log link and gaussian distribution

	Any NCEA Attainment		NCEA 3 Attainment		School Attendance		Tertiary Enrolment	
	**RR (95% CI)**	**ARR (95% CI)**	**RR (95% CI)**	**ARR (95% CI)**	**RR (95% CI)**	**ARR (95% CI)**	**RR (95% CI)**	**ARR (95% CI)**
T1D Status								
No	1 (reference)		1 (reference)		1 (reference)		1 (reference)	
Yes	0.916 (0.872, 0.962)	0.914 (0.87, 0.959)	0.741 (0.652, 0.843)	0.776 (0.688, 0.876)	0.701 (0.576, 0.854)	0.655 (0.526, 0.815)	0.808 (0.684, 0.954)	0.838 (0.721, 0.975)
Sex								
Female	1 (reference)		1 (reference)		1 (reference)		1 (reference)	
Male		0.960 (0.958, 0.963)		0.767 (0.763, 0.772)		1.125 (1.115, 1.136)		0.716 (0.711, 0.722)
Ethnicity								
Asian		1.051 (1.047, 1.056)		1.234 (1.222, 1.247)		1.272 (1.249, 1.295)		1.333 (1.314, 1.351)
EO		1.044 (1.040, 1.049)		1.018 (1.008, 1.028)		1.061 (1.043, 1.079)		1.067 (1.053, 1.082)
MELAA		0.968 (0.955, 0.982)		0.924 (0.896, 0.954)		0.994 (0.938, 1.052)		0.906 (0.868, 0.945)
Māori		0.940 (0.937, 0.943)		0.808 (0.800, 0.815)		0.716 (0.704, 0.728)		0.771 (0.761, 0.781)
Pacific		1.046 (1.041, 1.050)		1.008 (0.997, 1.020)		0.818 (0.800, 0.836)		0.961 (0.945, 0.977)
Deprivation								
1 (least deprived)	1 (reference)		1 (reference)		1 (reference)		1 (reference)	
2		0.989 (0.986, 0.991)		0.931 (0.924, 0.937)		0.959 (0.947, 0.971)		0.926 (0.918, 0.935)
3		0.972 (0.969, 0.975)		0.864 (0.857, 0.871)		0.914 (0.901, 0.926)		0.855 (0.846, 0.864)
4		0.952 (0.949, 0.955)		0.791 (0.784, 0.798)		0.861 (0.848, 0.875)		0.779 (0.770, 0.789)
5 (most deprived)		0.903 (0.899, 0.907)		0.696 (0.688, 0.704)		0.772 (0.758, 0.787)		0.648 (0.638, 0.658)
Rural residential location								
No	1 (reference)		1 (reference)		1 (reference)		1 (reference)	
Yes		1.010 (1.007, 1.013)		0.939 (0.931, 0.947)		1.063 (1.050, 1.077)		0.875 (0.864, 0.885)
Mother NZ born								
No	1 (reference)		1 (reference)		1 (reference)		1 (reference)	
Yes		1.022 (1.020, 1.025)		1.053 (1.046, 1.061)		1.043 (1.030, 1.056)		1.065 (1.055, 1.074)
Mother highest qual								

No qualification	1 (reference)	1 (reference)	1 (reference)	1 (reference)
School qualification	1.114 (1.107, 1.120)	1.429 (1.407, 1.452)	1.188 (1.162, 1.214)	1.522 (1.490, 1.555)
Tertiary certificate/diploma	1.132 (1.125, 1.138)	1.527 (1.503, 1.551)	1.199 (1.172, 1.226)	1.727 (1.690, 1.764)
University degree or higher	1.169 (1.163, 1.176)	1.828 (1.799, 1.856)	1.298 (1.270, 1.328)	2.213 (2.166, 2.261)
Mother's maternal age (years)				
<20	0.921 (0.915, 0.928)	0.688 (0.673, 0.703)	0.783 (0.761, 0.806)	0.633 (0.613, 0.654)
20-29	1 (reference)	1 (reference)	1 (reference)	1 (reference)
30-29	1.028 (1.026, 1.031)	1.192 (1.185, 1.200)	1.084 (1.073, 1.095)	1.212 (1.202, 1.221)
40-49	1.020 (1.013, 1.027)	1.193 (1.173, 1.213)	1.033 (1.000, 1.068)	1.249 (1.223, 1.275)
50+	0.904 (0.773, 1.058)	0.752 (0.520, 1.087)	0.986 (0.557, 1.746)	0.864 (0.507, 1.473)
Number of siblings				
0	1 (reference)	1 (reference)	1 (reference)	1 (reference)
1	1.018 (1.014, 1.021)	1.060 (1.050, 1.070)	1.085 (1.067, 1.103)	1.049 (1.036, 1.062)
2	1.007 (1.003, 1.011)	1.034 (1.024, 1.044)	1.070 (1.051, 1.089)	1.020 (1.007, 1.033)
3	0.976 (0.972, 0.981)	0.956 (0.944, 0.967)	0.999 (0.978, 1.021)	0.920 (0.906, 0.935)
4	0.931 (0.924, 0.937)	0.825 (0.809, 0.840)	0.877 (0.850, 0.906)	0.780 (0.760, 0.799)
5+	0.848 (0.841, 0.856)	0.707 (0.692, 0.723)	0.777 (0.750, 0.806)	0.628 (0.608, 0.647)
N	358,944	358,944	157,788	288,720

*Adjusted for birth year, sex, ethnicity, deprivation level, urban/rural profile of residence, maternal education level, maternal born in NZ status, maternal age, and number of siblings.

ESM Table 9: Unadjusted and adjusted risk ratios of educational outcomes on T1D without hospitalization for DKA/hypoglycaemia or early onset using complete-case generalized linear regression with a log link and gaussian distribution

	Any NCEA Attainment		NCEA 3 Attainment		School Attendance		Tertiary Enrolment	
	RR (95% CI)	ARR (95% CI)	RR (95% CI)	ARR (95% CI)	RR (95% CI)	ARR (95% CI)	RR (95% CI)	ARR (95% CI)
T1D Status								
No	1 (reference)		1 (reference)		1 (reference)		1 (reference)	
Yes	0.988 (0.964, 1.012)	0.979 (0.957, 1.001)	0.915 (0.857, 0.977)	0.906 (0.856, 0.960)	0.948 (0.857, 1.048)	0.965 (0.877, 1.061)	1.018 (0.94, 1.102)	0.954 (0.889, 1.024)
Sex								
Female	1 (reference)		1 (reference)		1 (reference)		1 (reference)	
Male		0.961 (0.958, 0.963)		0.767 (0.763, 0.772)		1.126 (1.115, 1.137)		0.716 (0.711, 0.722)
Ethnicity								
Asian		1.052 (1.047, 1.056)		1.235 (1.222, 1.248)		1.273 (1.250, 1.297)		1.333 (1.315, 1.351)
EO		1.044 (1.040, 1.049)		1.018 (1.008, 1.028)		1.062 (1.044, 1.080)		1.068 (1.054, 1.082)
MELAA		0.968 (0.955, 0.981)		0.923 (0.895, 0.953)		0.993 (0.937, 1.051)		0.906 (0.868, 0.945)
Māori		0.940 (0.937, 0.943)		0.808 (0.800, 0.815)		0.716 (0.705, 0.728)		0.771 (0.762, 0.781)
Pacific		1.046 (1.041, 1.050)		1.008 (0.996, 1.020)		0.819 (0.801, 0.837)		0.961 (0.945, 0.977)
Deprivation								
1 (least deprived)	1 (reference)		1 (reference)		1 (reference)		1 (reference)	
2		0.989 (0.986, 0.991)		0.931 (0.924, 0.937)		0.958 (0.946, 0.971)		0.926 (0.918, 0.935)
3		0.972 (0.969, 0.975)		0.864 (0.857, 0.871)		0.913 (0.901, 0.926)		0.856 (0.847, 0.865)
4		0.952 (0.949, 0.955)		0.791 (0.784, 0.798)		0.861 (0.848, 0.875)		0.779 (0.770, 0.789)
5 (most deprived)		0.903 (0.899, 0.907)		0.696 (0.688, 0.704)		0.773 (0.758, 0.787)		0.648 (0.638, 0.658)
Rural residential location								
No	1 (reference)		1 (reference)		1 (reference)		1 (reference)	
Yes		1.010 (1.007, 1.013)		0.939 (0.931, 0.946)		1.063 (1.049, 1.077)		0.874 (0.864, 0.884)
Mother NZ born								
No	1 (reference)		1 (reference)		1 (reference)		1 (reference)	
Yes		1.022 (1.020, 1.025)		1.053 (1.046, 1.061)		1.043 (1.029, 1.056)		1.065 (1.055, 1.074)
Mother highest qual								

No qualification	1 (reference)	1 (reference)	1 (reference)	1 (reference)
School qualification	1.114 (1.107, 1.120)	1.429 (1.407, 1.451)	1.188 (1.162, 1.214)	1.520 (1.488, 1.553)
Tertiary certificate/diploma	1.132 (1.125, 1.138)	1.527 (1.503, 1.551)	1.199 (1.172, 1.226)	1.724 (1.687, 1.762)
University degree or higher	1.169 (1.163, 1.176)	1.827 (1.799, 1.856)	1.298 (1.270, 1.327)	2.210 (2.164, 2.258)
Mother's maternal age (years)				
<20	0.921 (0.915, 0.928)	0.687 (0.673, 0.703)	0.783 (0.761, 0.806)	0.633 (0.613, 0.654)
20-29	1 (reference)	1 (reference)	1 (reference)	1 (reference)
30-29	1.028 (1.026, 1.031)	1.193 (1.186, 1.2)00	1.084 (1.073, 1.095)	1.212 (1.202, 1.222)
40-49	1.021 (1.013, 1.028)	1.194 (1.174, 1.214)	1.034 (1.001, 1.069)	1.251 (1.225, 1.277)
50+	0.905 (0.773, 1.058)	0.752 (0.520, 1.087)	0.986 (0.556, 1.746)	0.864 (0.507, 1.473)
Number of siblings				
0	1 (reference)	1 (reference)	1 (reference)	1 (reference)
1	1.017 (1.014, 1.021)	1.059 (1.050, 1.069)	1.085 (1.066, 1.103)	1.049 (1.036, 1.062)
2	1.007 (1.003, 1.011)	1.033 (1.023, 1.043)	1.069 (1.050, 1.088)	1.020 (1.007, 1.033)
3	0.976 (0.972, 0.981)	0.955 (0.944, 0.967)	0.999 (0.978, 1.020)	0.920 (0.906, 0.935)
4	0.931 (0.924, 0.937)	0.825 (0.809, 0.840)	0.878 (0.851, 0.906)	0.779 (0.760, 0.799)
5+	0.848 (0.841, 0.856)	0.707 (0.692, 0.722)	0.778 (0.750, 0.806)	0.628 (0.609, 0.648)
N	359,562	359,562	158,031	289,251

*Adjusted for birth year, sex, ethnicity, deprivation level, urban/rural profile of residence, maternal education level, maternal born in NZ status, maternal age, and number of siblings.

ESM Table 10: Unadjusted and adjusted risk ratios of educational outcomes on T1D status using complete-case generalized linear regression with a log link and gaussian distribution among Māori

	Any NCEA Attainment		NCEA 3 Attainment		School Attendance		Tertiary Enrolment	
	**RR (95% CI)**	**ARR (95% CI)**	**RR (95% CI)**	**ARR (95% CI)**	**RR (95% CI)**	**ARR (95% CI)**	**RR (95% CI)**	**ARR (95% CI)**
T1D Status								
No	1 (reference)		1 (reference)		1 (reference)		1 (reference)	
Yes	0.986 (0.942, 1.033)	0.966 (0.922, 1.012)	0.861 (0.753, 0.984)	0.830 (0.722, 0.954)	1.085 (0.916, 1.286)	1.137 (0.962, 1.345)	1.06 (0.892, 1.261)	0.935 (0.786, 1.113)
Sex								
Female	1 (reference)		1 (reference)		1 (reference)		1 (reference)	
Male		0.943 (0.938, 0.949)		0.722 (0.711, 0.734)		1.204 (1.173, 1.237)		0.611 (0.597, 0.625)
Deprivation								
1 (least deprived)	1 (reference)		1 (reference)		1 (reference)		1 (reference)	
2		0.983 (0.975, 0.991)		0.893 (0.872, 0.914)		0.942 (0.903, 0.982)		0.878 (0.851, 0.906)
3		0.955 (0.947, 0.963)		0.828 (0.809, 0.848)		0.838 (0.803, 0.875)		0.772 (0.747, 0.796)
4		0.926 (0.918, 0.934)		0.737 (0.719, 0.755)		0.753 (0.721, 0.786)		0.668 (0.647, 0.690)
5 (most deprived)		0.863 (0.855, 0.870)		0.639 (0.624, 0.655)		0.619 (0.593, 0.646)		0.509 (0.492, 0.527)
Rural residential location								
No	1 (reference)		1 (reference)		1 (reference)		1 (reference)	
Yes		1.026 (1.019, 1.034)		0.950 (0.93, 0.971)		1.067 (1.031, 1.104)		0.881 (0.853, 0.910)
Mother NZ born								
No	1 (reference)		1 (reference)		1 (reference)		1 (reference)	
Yes		1.006 (0.995, 1.017)		1.030 (1.001, 1.059)		1.046 (0.993, 1.101)		1.055 (1.017, 1.094)
Mother highest qual								
No qualification	1 (reference)		1 (reference)		1 (reference)		1 (reference)	
School qualification		1.116 (1.103, 1.129)		1.474 (1.422, 1.527)		1.166 (1.108, 1.227)		1.621 (1.538, 1.708)
Tertiary certificate/diploma		1.162 (1.149, 1.177)		1.635 (1.578, 1.695)		1.163 (1.103, 1.225)		1.876 (1.780, 1.977)
University degree or higher		1.249 (1.234, 1.264)		2.156 (2.080, 2.235)		1.295 (1.228, 1.365)		2.779 (2.638, 2.927)

Mother's maternal age (years)				
<20	0.942 (0.932, 0.952)	0.779 (0.755, 0.803)	0.787 (0.751, 0.825)	0.697 (0.664, 0.732)
20-29	1 (reference)	1 (reference)	1 (reference)	1 (reference)
30-29	1.039 (1.033, 1.045)	1.236 (1.217, 1.256)	1.136 (1.104, 1.169)	1.286 (1.258, 1.314)
40-49	1.036 (1.014, 1.058)	1.239 (1.178, 1.304)	0.989 (0.888, 1.101)	1.239 (1.154, 1.330)
50+	0.923 (0.686, 1.243)	0.400 (0.098, 1.630)	0.386 (0.057, 1.843)	0.728 (0.216, 2.457)
Number of siblings				
0	1 (reference)	1 (reference)	1 (reference)	1 (reference)
1	1.021 (1.011, 1.032)	1.046 (1.017, 1.076)	1.110 (1.053, 1.170)	1.026 (0.989, 1.064)
2	0.993 (0.982, 1.003)	0.969 (0.941, 0.997)	1.031 (0.977, 1.087)	0.927 (0.893, 0.963)
3	0.949 (0.938, 0.960)	0.862 (0.835, 0.890)	0.909 (0.857, 0.963)	0.791 (0.758, 0.825)
4	0.897 (0.884, 0.910)	0.723 (0.695, 0.752)	0.756 (0.705, 0.811)	0.634 (0.600, 0.670)
5+	0.807 (0.795, 0.819)	0.611 (0.586, 0.636)	0.667 (0.621, 0.716)	0.513 (0.484, 0.544)
N	107,310	107,310	45,741	88,572

*Adjusted for birth year, sex, deprivation level, urban/rural profile of residence, maternal education level, maternal born in NZ status, maternal age, and number of siblings.

ESM Table 11: Unadjusted and adjusted risk ratios of educational outcomes on T1D status using complete-case generalized linear regression with a log link and gaussian distribution among Pacific peoples

	Any NCEA Attainment		NCEA 3 Attainment		School Attendance		Tertiary Enrolment	
	RR (95% CI)	ARR (95% CI)	RR (95% CI)	ARR (95% CI)	RR (95% CI)	ARR (95% CI)	RR (95% CI)	ARR (95% CI)
T1D Status								
No	1 (reference)		1 (reference)		1 (reference)		1 (reference)	
Yes	0.939 (0.884, 0.998)	0.936 (0.881, 0.995)	0.947 (0.817, 1.098)	0.963 (0.830, 1.117)	1.015 (0.816, 1.264)	1.034 (0.821, 1.303)	0.978 (0.763, 1.254)	0.876 (0.669, 1.147)
Sex								
Female	1 (reference)		1 (reference)		1 (reference)		1 (reference)	
Male		0.947 (0.939, 0.954)		0.762 (0.746, 0.779)		1.200 (1.154, 1.248)		0.590 (0.571, 0.610)
Deprivation								
1 (least deprived)	1 (reference)		1 (reference)		1 (reference)		1 (reference)	
2		0.991 (0.977, 1.006)		0.891 (0.854, 0.929)		0.895 (0.823, 0.972)		0.877 (0.829, 0.928)
3		0.973 (0.959, 0.987)		0.838 (0.804, 0.872)		0.785 (0.722, 0.853)		0.780 (0.738, 0.825)
4		0.952 (0.939, 0.965)		0.783 (0.754, 0.813)		0.800 (0.742, 0.862)		0.746 (0.709, 0.786)
5 (most deprived)		0.909 (0.898, 0.921)		0.722 (0.698, 0.748)		0.752 (0.703, 0.805)		0.601 (0.573, 0.632)
Rural residential location								
No	1 (reference)		1 (reference)		1 (reference)		1 (reference)	
Yes		0.982 (0.963, 1.002)		0.850 (0.801, 0.903)		0.987 (0.891, 1.093)		0.798 (0.729, 0.873))
Mother NZ born								
No	1 (reference)		1 (reference)		1 (reference)		1 (reference)	
Yes		1.071 (1.063, 1.080)		1.167 (1.141, 1.194)		1.265 (1.213, 1.319)		1.211 (1.173, 1.250)
Mother highest qual								
No qualification	1 (reference)		1 (reference)		1 (reference)		1 (reference)	
School qualification		1.072 (1.057, 1.087)		1.251 (1.203, 1.301)		1.168 (1.093, 1.248)		1.420 (1.336, 1.510)
Tertiary certificate/diploma		1.082 (1.066, 1.098)		1.291 (1.239, 1.346)		1.157 (1.077, 1.243)		1.549 (1.454, 1.651)
University degree or higher		1.144 (1.128, 1.161)		1.592 (1.527, 1.658)		1.333 (1.239, 1.434)		2.153 (2.022, 2.292)

Mother's maternal age (years)				
<20	0.918 (0.903, 0.933)	0.741 (0.707, 0.776)	0.798 (0.740, 0.861)	0.680 (0.631, 0.733)
20-29	1 (reference)	1 (reference)	1 (reference)	1 (reference)
30-29	1.021 (1.013, 1.030)	1.119 (1.094, 1.145)	1.080 (1.034, 1.128)	1.143 (1.107, 1.181)
40-49	1.027 (1.003, 1.051)	1.116 (1.044, 1.193)	1.033 (0.902, 1.182)	1.194 (1.098, 1.299)
50+	0.818 (0.635, 1.054)	0.868 (0.588, 1.283)	1.566 (0.920, 2.665)	0.546 (0.179, 1.661)
Number of siblings				
0	1 (reference)	1 (reference)	1 (reference)	1 (reference)
1	1.008 (0.993, 1.023)	1.033 (0.992, 1.077)	1.132 (1.044, 1.226)	1.023 (0.968, 1.082)
2	1.003 (0.989, 1.018)	1.002 (0.962, 1.043)	1.076 (0.993, 1.165)	0.989 (0.935, 1.046)
3	0.985 (0.970, 1.000)	0.935 (0.895, 0.975)	1.013 (0.932, 1.100)	0.851 (0.801, 0.903)
4	0.961 (0.945, 0.978)	0.831 (0.791, 0.874)	0.892 (0.812, 0.980)	0.764 (0.712, 0.819)
5+	0.893 (0.876, 0.909)	0.75 (0.713, 0.788)	0.776 (0.707, 0.852)	0.636 (0.591, 0.685)
N	41,079	41,079	19,701	32,814

*Adjusted for birth year, sex, deprivation level, urban/rural profile of residence, maternal education level, maternal born in NZ status, maternal age, and number of siblings.

ESM Table 12: Sensitivity 1, unadjusted and adjusted risk ratios of educational outcomes on T1D status using complete-case generalized linear regression with a log link and gaussian distribution with missing educational outcome data set to no attainment (for the any NCEA and NCEA 3 outcomes) and for no regular attendance (for the school attendance outcome)

	**Any NCEA Attainment**		**NCEA 3 Attainment**		**School Attendance**	
	**RR (95% CI)**	**ARR (95% CI)**	**RR (95% CI)**	**ARR (95% CI)**	**RR (95% CI)**	**ARR (95% CI)**
T1D Status						
No	1 (reference)		1 (reference)		1 (reference)	
Yes	1.002 (0.983, 1.022)	0.981 (0.963, 0.999)	0.912 (0.868, 0.959)	0.887 (0.848, 0.928)	0.920 (0.855, 0.990)	0.920 (0.856, 0.989)
Sex						
Female	1 (reference)		1 (reference)		1 (reference)	
Male		0.954 (0.952, 0.956)		0.765 (0.760, 0.769)		1.125 (1.113, 1.136)
Ethnicity						
Asian		1.048 (1.042, 1.054)		1.224 (1.211, 1.238)		1.265 (1.240, 1.290)
EO		1.068 (1.063, 1.073)		1.035 (1.025, 1.046)		1.073 (1.054, 1.093)
MELAA		0.938 (0.922, 0.953)		0.901 (0.872, 0.931)		1.005 (0.945, 1.069)
Māori		0.928 (0.925, 0.932)		0.801 (0.793, 0.808)		0.722 (0.709, 0.734)
Pacific		1.048 (1.042, 1.053)		1.007 (0.995, 1.019)		0.857 (0.838, 0.877)
Deprivation						
1 (least deprived)	1 (reference)		1 (reference)		1 (reference)	
2		0.987 (0.984, 0.990)		0.929 (0.922, 0.936)		0.990 (0.976, 1.004)
3		0.967 (0.964, 0.970)		0.860 (0.853, 0.867)		0.959 (0.945, 0.973)
4		0.941 (0.938, 0.945)		0.784 (0.776, 0.791)		0.900 (0.885, 0.916)
5 (most deprived)		0.878 (0.874, 0.883)		0.680 (0.672, 0.688)		0.806 (0.790, 0.823)
Rural residential location						
No	1 (reference)		1 (reference)		1 (reference)	
Yes		1.009 (1.005, 1.012)		0.938 (0.93, 0.946)		1.067 (1.052, 1.082)
Mother NZ born						
No	1 (reference)		1 (reference)		1 (reference)	
Yes		1.023 (1.019, 1.026)		1.053 (1.045, 1.06)		1.041 (1.027, 1.055)
Mother highest qual						

No qualification	1 (reference)	1 (reference)	1 (reference)
School qualification	1.128 (1.121, 1.135)	1.450 (1.427, 1.473)	1.186 (1.159, 1.214)
Tertiary certificate/diploma	1.148 (1.140, 1.155)	1.549 (1.524, 1.574)	1.193 (1.165, 1.222)
University degree or higher	1.191 (1.184, 1.198)	1.858 (1.828, 1.888)	1.257 (1.228, 1.287)
Mother's maternal age (years)			
<20	0.906 (0.898, 0.913)	0.674 (0.660, 0.690)	0.776 (0.753, 0.799)
20-29	1 (reference)	1 (reference)	1 (reference)
30-29	1.030 (1.027, 1.033)	1.194 (1.187, 1.201)	1.065 (1.053, 1.076)
40-49	1.008 (1.000, 1.017)	1.180 (1.160, 1.201)	1.001 (0.965, 1.037)
50+	0.857 (0.713, 1.029)	0.712 (0.478, 1.059)	1.004 (0.558, 1.807)
Number of siblings			
0	1 (reference)	1 (reference)	1 (reference)
1	1.027 (1.023, 1.032)	1.069 (1.059, 1.079)	1.117 (1.096, 1.138)
2	1.017 (1.013, 1.022)	1.045 (1.034, 1.055)	1.097 (1.076, 1.119)
3	0.979 (0.974, 0.985)	0.961 (0.949, 0.973)	1.010 (0.987, 1.034)
4	0.924 (0.917, 0.932)	0.822 (0.806, 0.838)	0.859 (0.831, 0.888)
5+	0.829 (0.821, 0.837)	0.693 (0.677, 0.708)	0.701 (0.674, 0.728)
N	377,988	377,988	184,023

*Adjusted for birth year, sex, ethnicity, deprivation level, urban/rural profile of residence, maternal education level, maternal born in NZ status, maternal age, and number of siblings.

ESM Table 13: Sensitivity 2, unadjusted and adjusted risk ratios of educational outcomes on T1D status using complete-case generalized linear regression with a log link and gaussian distribution excluding individuals who had ever been enrolled in a specialist school

	Any NCEA Attainment		NCEA 3 Attainment		School Attendance		Tertiary Enrolment	
	RR (95% CI)	ARR (95% CI)	RR (95% CI)	ARR (95% CI)	RR (95% CI)	ARR (95% CI)	RR (95% CI)	ARR (95% CI)
T1D Status								
No	1 (reference)		1 (reference)		1 (reference)		1 (reference)	
Yes	0.983 (0.965, 1)	0.974 (0.958, 0.991)	0.897 (0.854, 0.941)	0.884 (0.846, 0.925)	0.916 (0.854, 0.983)	0.913 (0.853, 0.978)	0.986 (0.925, 1.052)	0.929 (0.877, 0.984)
Sex								
Female	1 (reference)		1 (reference)		1 (reference)		1 (reference)	
Male		0.961 (0.959, 0.963)		0.765 (0.761, 0.769)		1.117 (1.106, 1.127)		0.716 (0.71, 0.721)
Ethnicity								
Asian		1.051 (1.047, 1.056)		1.233 (1.22, 1.246)		1.273 (1.25, 1.297)		1.332 (1.314, 1.351)
EO		1.044 (1.04, 1.049)		1.021 (1.011, 1.031)		1.068 (1.05, 1.086)		1.067 (1.053, 1.081)
MELAA		0.967 (0.954, 0.981)		0.924 (0.896, 0.953)		0.991 (0.936, 1.05)		0.906 (0.868, 0.945)
Māori		0.941 (0.938, 0.944)		0.809 (0.802, 0.816)		0.717 (0.706, 0.729)		0.772 (0.762, 0.782)
Pacific		1.045 (1.04, 1.05)		1.008 (0.997, 1.02)		0.817 (0.799, 0.835)		0.961 (0.946, 0.977)
Deprivation								
1 (least deprived)	1 (reference)		1 (reference)		1 (reference)		1 (reference)	
2		0.99 (0.987, 0.992)		0.932 (0.926, 0.938)		0.959 (0.947, 0.972)		0.927 (0.918, 0.935)
3		0.973 (0.97, 0.976)		0.867 (0.86, 0.874)		0.917 (0.904, 0.929)		0.856 (0.847, 0.865)
4		0.954 (0.951, 0.957)		0.794 (0.787, 0.801)		0.866 (0.853, 0.88)		0.78 (0.771, 0.79)
5 (most deprived)		0.905 (0.901, 0.909)		0.699 (0.691, 0.707)		0.777 (0.762, 0.792)		0.649 (0.639, 0.659)
Rural residential location								
No	1 (reference)		1 (reference)		1 (reference)		1 (reference)	
Yes		1.01 (1.007, 1.013)		0.938 (0.931, 0.946)		1.062 (1.048, 1.075)		0.874 (0.864, 0.884)
Mother NZ born								
No	1 (reference)		1 (reference)		1 (reference)		1 (reference)	
Yes		1.022 (1.019, 1.025)		1.053 (1.046, 1.060)		1.041 (1.028, 1.055)		1.064 (1.055, 1.074)
Mother highest qual								

No qualification	1 (reference)	1 (reference)	1 (reference)	1 (reference)
School qualification	1.112 (1.105, 1.118)	1.425 (1.403, 1.447)	1.187 (1.161, 1.214)	1.518 (1.486, 1.551)
Tertiary certificate/diploma	1.129 (1.123, 1.136)	1.521 (1.498, 1.546)	1.199 (1.172, 1.226)	1.721 (1.684, 1.759)
University degree or higher	1.166 (1.159, 1.172)	1.820 (1.792, 1.848)	1.300 (1.271, 1.329)	2.206 (2.160, 2.254)
Mother's maternal age (years)				
<20	0.924 (0.917, 0.930)	0.692 (0.677, 0.707)	0.780 (0.757, 0.803)	0.634 (0.614, 0.655)
20-29	1 (reference)	1 (reference)	1 (reference)	1 (reference)
30-29	1.028 (1.026, 1.030)	1.191 (1.184, 1.198)	1.083 (1.072, 1.094)	1.212 (1.202, 1.222)
40-49	1.021 (1.014, 1.028)	1.197 (1.177, 1.216)	1.035 (1.002, 1.070)	1.252 (1.226, 1.278)
50+	0.917 (0.781, 1.075)	0.819 (0.583, 1.150)	1.097 (0.594, 2.026)	0.800 (0.447, 1.433)
Number of siblings				
0	1 (reference)	1 (reference)	1 (reference)	1 (reference)
1	1.017 (1.013, 1.020)	1.057 (1.048, 1.067)	1.082 (1.064, 1.100)	1.048 (1.036, 1.061)
2	1.007 (1.003, 1.010)	1.032 (1.022, 1.042)	1.067 (1.048, 1.086)	1.019 (1.006, 1.033)
3	0.976 (0.971, 0.981)	0.955 (0.944, 0.967)	0.997 (0.976, 1.019)	0.920 (0.905, 0.935)
4	0.931 (0.924, 0.938)	0.825 (0.810, 0.841)	0.879 (0.851, 0.907)	0.779 (0.760, 0.799)
5+	0.849 (0.842, 0.856)	0.709 (0.694, 0.725)	0.777 (0.749, 0.806)	0.628 (0.609, 0.647)
N	356,124	356,124	155,070	288,108

*Adjusted for birth year, sex, ethnicity, deprivation level, urban/rural profile of residence, maternal education level, maternal born in NZ status, maternal age, and number of siblings.
